# Declines in marathon performance: Sex differences in elite and recreational athletes

**DOI:** 10.1371/journal.pone.0172121

**Published:** 2017-02-10

**Authors:** Gerald S. Zavorsky, Kelly A. Tomko, James M. Smoliga

**Affiliations:** 1 Department Respiratory Therapy, Georgia State University, Atlanta, Georgia, United States of America; 2 University of Bridgeport, Bridgeport, Connecticut, United States of America; 3 Department of Physical Therapy, High Point University, High Point, North Carolina, United States of America; University of Sydney, AUSTRALIA

## Abstract

The first aim of this study was to determine the age group at which marathon performance declines in top male and female runners and to compare that to the runners of average ability. Another aim of this of this study was to examine the age-related yearly decline in marathon performance between age group winners and the average marathon finisher. Data from the New York (NYC), Boston, and Chicago marathons from 2001–2016 were analyzed. Age, sex, and location were used in multiple linear regression models to determine the rate of decline in marathon times. Winners of each age group were assessed in 5-year increments from 16 through 74 years old (n = 47 per age group). The fastest times were between 25–34 years old, with overall champion males at 28.3 years old, and overall champion females at 30.8 years old (*p* = 0.004). At 35 years of age up to 74 years of age, female age group winners had a faster yearly decline in marathon finishing times compared to male age group winners, irrespective of marathon location [women = (min:sec) 2:33 per year, n = 336; men = 2:06 per year, n = 373, *p* < 0.01]. The median times between each age group only slowed beginning at 50 years old, thereafter the decline was similar between both men and women (women = 2:36, n = 140; men = 2:57, n = 150, *p* = 0.11). The median times were fastest at Boston and similar between Chicago and NYC. In conclusion, the rate of decline at 35 years old up to 74 years old is roughly linear (adjusted r^2^ = 0.88, *p* < 0.001) with female age group winners demonstrating 27 s per year greater decline per year compared to male age group winners.

## Introduction

The relationship between age and running time in elite marathoners is U-shaped, where between 15 and 25 years old, marathon times get faster, and then after about 35 years of age, marathon times get slower [1–4]. Since ∼40 to 77% of the variation in marathon performance is accounted for by differences in maximal oxygen consumption [5–10], then the 1–2% decline in maximal oxygen consumption after 25–35 years of age [11] is partially responsible for the age-related decline in marathon performance. Aging studies show that peak endurance performance is maintained until ∼35 years of age [3, 4, 12], after which maximal oxygen consumption, lactate threshold, and exercise economy all progressively decline [11, 13]. As well, a reduction in overall exercise training stimulus tends to decline with age, too [13].

Over the past 16 years, the vast majority of the overall marathon winners come from the 25–29 and 30 to 34 years old age groups (Tables 1 and 2). Studies demonstrate that at ∼35 years of age, the “slow-down” of marathon finishing times is relatively linear in elite runners with a ∼2.5% per year rate of decline [1]. However, the annual rate of decline in marathon performance (minutes:seconds) in age-group winners compared to average finishers is not known. Some studies use only the mean finishing times for a given age range to determine the rate of decline [14, 15], while others only used data from one or two marathons spread over one to two years [1, 2] or one marathon spread over many years [3, 16, 17]. In the former studies [14, 15] the lack of a decline in marathon times between 20–49 or 20–54 years is due to combining all the finishers, fast and slow, together in each age group and generating a mean finishing time for each age group. Combining all runners together not help in deciphering the annual rate of decline between the top runners compared to the average finishers as the mean data is influenced by slower and faster runners, extreme outliers, and the number of finishers per age group. As such, the slowest and fastest finishers in each age group cancel out, and a meaningful understanding of the decline in marathon performance with aging cannot be ascertained. The studies that analyzed overall winners and age group winners together with the rest of the finishers [14, 15] does not allow one to separately compare the rate of decline between the top runners versus the average runner, as we sought to do. In other studies, a quadratic function was used to fit the data [1–3], but we feel that a simple linear model could also fit the age-related decline in marathon performance beginning at 35 years old. The first aim of this study was to determine the age group at which marathon performance declines in top male and female runners and to compare that to the runners of average ability (those that finish in the middle of their age group). Based on the data from Leyk and colleagues who examined the top 10 overall finishers from several German marathons between 2003–2005 [14], it is our hypothesis that the decline in marathon performance would begin at about 35 years old (i.e. the 35–39 years age group). For the runners of average ability, the age-related decline in performance would begin later, perhaps at around 50 years old, as demonstrated elsewhere [14].

**Table 1:** Overall male marathon champions and their respective ages.

	Boston		New York City		Chicago	
Year	Name	age	Name	age	Name	Age
2001	Lee, Bong-Ju	30.5	Tesfaye, Jifar	25.6	Kimondiu, Ben	23.9
2002	Rop, Rodgers	26.2	Rop, Rodgers	26.7	Khannouchi, Khalid	30.8
2003	Cheruiyot, Robert Kipkoech	24.6	Lel, Martin	25.0	Rutto, Evans	25.5
2004	Cherigat, Timothy	27.3	Ramaala, Hendrik	32.8	Rutto, Evans	26.5
2005	Negussie, Hailu	27.0	Tergat, Paul	36.4	Limo, Felix	25.2
2006	Cheruiyot, Robert Kipkoech	27.6	Gomes Dos Santos, Marilson	29.3	Cheruiyot, Robert Kipkoech	28.1
2007	Cheruiyot, Robert Kipkoech	28.6	Lel, Martin	29.0	Ivuti, Patrick	29.2
2008	Cheruiyot, Robert Kipkoech	29.6	Gomes Dos Santos, Marilson	31.3	Cheruiyot, Evans	26.4
2009	Merga, Deriba	28.5	Keflezighi, Meb	34.5	Wanjiru, Sammy	22.9
2010	Cheruiyot, Robert Kipkoech	31.6	Gebremariam, Gebre	26.2	Wanjiru, Sammy	23.9
2011	Mutai, Geoffrey	29.6	Mutai, Geoffrey	30.1	Mosop, Moses	26.3
2012	Korir, Wesley	29.4	The marathon was not run		Kebede, Tsegaye	25.8
2013	Desisa, Lelisa	23.3	Mutai, Geoffrey	32.1	Kimetto, Dennis	29.8
2014	Keflezighi, Meb	39.0	Kipsang, Wilson	32.7	Kipchoge, Eliud	30.0
2015	Desisa, Lelisa	25.3	Biwott, Stanley	29.6	Chumba, Dickson	29.0
2016	Hayle, Lemi Berhanu	21.6	Ghebreslassie, Ghirmay	21.0	Kirui, Abel	34.4
Average		28.1		29.5		27.4
SD		4.0		4.1		3.0
Min		21.6		21.0		22.9
Max		39.0		36.4		34.4

The last names are present first, followed by the first names.

**Table 2:** Overall female marathon champions and their respective ages.

	Boston		New York City		Chicago	
Year	Name	age	Name	age	Name	Age
2001	Ndereba, Catherine	28.8	Okayo, Margaret	25.5	Nderaba, Catherine	29.2
2002	Okayo, Margaret	25.9	Chepchumba, Joyce	32.0	Radcliffe, Paula	28.8
2003	Zakharova, Svetlana	32.6	Okayo, Margaret	27.4	Zakharova, Svetlana	33.1
2004	Ndereba, Catherine	31.8	Radcliffe, Paula	30.9	Tomescu-Dita, Constantina	34.7
2005	Ndereba, Catherine	32.8	Prokopcuka, Jelena	29.2	Kastor, Deena	32.7
2006	Jeptoo, Rita	25.2	Prokopcuka, Jelena	30.1	Adere, Berhane	32.9
2007	Grigoryeva, Lidiya	33.3	Radcliffe, Paula	33.9	Adere, Berhane	33.9
2008	Tune, Dire	22.9	Radcliffe, Paula	34.9	Grigoryeva, Lidiya	34.8
2009	Kosgei, Salina	32.5	Tulu, Derartu	37.6	Mikitenko, Irina	37.2
2010	Erkesso, Teyba	27.5	Kiplagat, Edna	31.0	Baysa, Astede	23.5
2011	Kilel, Caroline	30.1	Dado, Firehiwot	27.8	Dibaba, Ejegayehu	29.8
2012	Cherop, Sharon	28.1	The marathon was not run		Baysa, Astede	25.5
2013	Jeptoo, Rita	32.2	Jeptoo, Priscah	29.4	Jeptoo, Rita	32.7
2014	Jeptoo, Rita	33.2	Keitany, Mary	32.8	Jeptoo, Rita	33.7
2015	Rotich, Caroline	31.0	Keitany, Mary	33.8	Kiplagat, Florence	28.6
2016	Baysa, Atsede	29.0	Keitany, Mary	34.8	Kiplagat, Florence	29.6
Average		29.8		31.4		31.3
SD		3.2		3.3		3.6
Min		22.9		25.5		23.5
Max		33.3		37.6		37.2

The last names are present first, followed by the first names.

Other studies only focus on increasing participation rates or performances in older runners [17–20], or pacing strategies [21, 22] and do not examine declining performances across a wide range of ages. Thus, another aim of this study was to examine the age-related decline in marathon performance between age group winners and the average marathon finisher (the median running time in his/her age group). Based on the data by Lehto and colleagues [3], it was our hypothesis that the decline in marathon performance would be fairly linear, with the age-group winners slowing down at a faster rate compared to the average finisher. Fairly linear means that the multiple linear regression model would have an R^2^ of at least 0.8.

There are several case studies and original research papers that look at the effects of aging on marathon performance [1–4, 12, 14–18, 23–27]. However, there is a combination of several aspects of this study which makes it novel. First, we chose a recent 16-year period of analyses (2001–2016) to account for year to year differences in the weather, which affects finishing times [28, 29]. Some studies do not do this [1, 2]. Second, we combined data from New York City, Chicago, and Boston Marathons because these three marathons have the most participants in North America [30]. In fact, about ∼116,000 runners ran the New York City, Chicago, and Boston Marathons combined in 2016, alone. None of the previous studies used data from all three marathons combined up to 2016.

## Methods

Institutional Review Board (IRB) approval was sought at Georgia State University (IRB number: H16184, Reference number: 336136) for the use of publically available data on marathon finishing times from Boston, New York City (NYC), and Chicago marathons between 2001 and 2016. The IRB determined that this research study was not human subjects’ research. Thus we were allowed to proceed without IRB oversight.

As such, data mining was performed by searching publically available websites for the Boston, NYC, and Chicago marathons. For each finisher, we obtained the sex, marathon finish time, year of the race, age, age group, and the city which the marathon was run. The sample included all participants from each of the 12 age groups (16–19; 20–24; 25–29; 30–34; 35–39; 40–44; 45–49; 50–54; 55–59; 60–64; 65–69; 70–74 years old) for each year between 2001 and 2016.

### Statistical analysis

First, to compare the age difference between the sexes for the overall champion runners from each marathon (listed in Tables 1 and 2), we used an independent *t*-test as the data was normally distributed. To determine whether marathon city influenced finish time in elite and recreational runners, a Kruskal-Wallis one-way analysis of variance (ANOVA) by ranks was used to compare the winning and median finishing times between the three cities when all age groups were placed together, and males and females were placed together. This type of ANOVA was used since the finishing times within each city were not normally distributed. If there was an overall significant difference in the ANOVA, then a Bonferroni correction was used to adjust the *p*-value for multiple tests. The effect size was then calculated as the *r*, which is the *z*-score divided by the square root of the sample size. Small, medium, and large effect sizes, were defined as *r* = 0.1, 0.3, and 0.5, respectively.

To determine differences in finishing time between age group categories in elite and recreational runners, a Kruskal-Wallis one-way analysis of variance (ANOVA) by ranks was used to compare finishing times among the twelve different age groups within each sex. This type of ANOVA was used since the finishing times within some age groups were not normally distributed. If and when the ANOVA was found to have overall statistical significance (*p* < 0.05), a stepped procedure (homogenous subsets) was used to determine where the significant differences were between the age groups. For this procedure, the *p*-value is adjusted overall, across all tests, and the type I error rate remains at 5%. This method does not compare every age group with every other age group (it does not perform all pairwise comparisons), so that the adjustment of *p*-values does not need to be so strict because there are not as many comparisons with the data, but rather selects age groups that are similar and places them together in subsets. The procedure that SPSS uses (stepped-procedure) begins by ordering the age groups based on the sum of ranks from smallest to largest. If there are ties, the median is used to decide the order. Step one of this procedure is to see whether the first ranked age group is the same as the second and if so, then the third age group is added to see if its ranking is statistically the same as the first two age groups. If yes, then the fourth age group is put together with the first three. At any point statistical significance is found, then the procedure is stopped, and the last age group that was included is now is carried into the next step, and the groups that are not carried forward is a subset (they are essentially the same). In step two, the process is repeated over again (see the detailed procedure on pages 238–239 and pages 243–246 of the following textbook [31]). We decided on a two-tailed type I probability level of 0.05.

To determine the rate of decline in marathon performance with age, multiple linear regression using the stepwise procedure was conducted to determine which independent variables [age, sex (0 = female, 1 = male), marathon location (Boston, Chicago, NYC)] were predictors of marathon finish time. Separate regression models were made for age group winners and for the average finisher (the median finishing times). Since regression is very sensitive to extreme cases, outliers were removed. Data were screened by identifying outliers. Any outlier, defined as ≥ 3.0 standard deviation of the residuals were eliminated. A plot was created between the standardized residuals (y-axis) and standardized predicted values (x-axis) to see if the values were consistently spread out, which would indicate normality and homoscedasticity. We also examined the probability-probability plot of the regression standardized residuals. Then curve fitting was used to determine whether the rate of decline in marathon performance was a linear or quadratic function.

To compare the rate of decline in marathon finishing times with age between men and women age group winners, we compared slopes by calculating the test statistic as *z*-scores. Thus, *z* = age rate decline in age group male winners (slope b_1_) minus age rate decline in age group female winners (slope b_2_) divided by the standard error of the difference between the two slopes, so *z* = (b_1_–b_2_) ÷ (S_b1-b2_). The standard error of the difference between the two slopes (S_b1-b2_) is calculated by taking the square root of (S_b1_^2^+S_b2_^2^), where S_b1_^2^ is the standard error of the slope of the age group male winners, squared; and S_b2_^2^ is the standard error of the slope of the age group female winners, squared. To compare the rate of decline between the average finisher for the males and the average finisher of the females, we also compared slopes by calculating the test statistic as z-scores. A z-score of ± 1.96 (two-tailed) was considered statistically significant. Statistical analyses were performed using IBM SPSS version 21.0, Chicago, IL.

## Results

### Missing data

**Fig 1:**
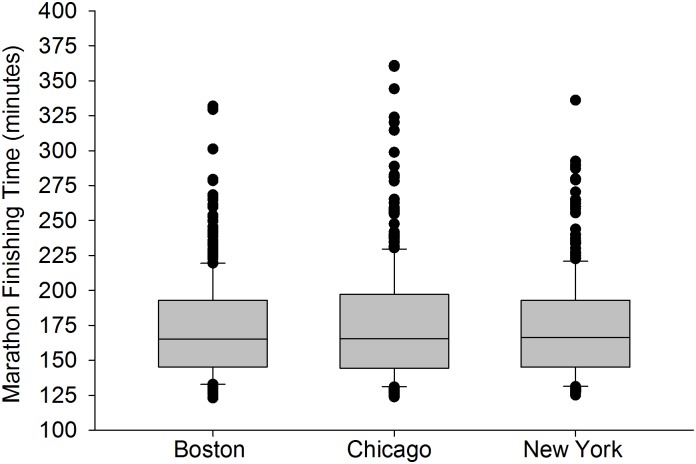
The winning finishing times combined across all age categories and both sexes for Boston (n = 382), Chicago (n = 387), and New York (n = 356) City Marathons between 2001 and 2016. Each box represents the 25^th^ (bottom border) 50^th^ (middle border), and 75^th^ (top border) percentiles. The whiskers (error bars) above and below each box represent the 90^th^ and 10^th^ percentiles, respectively. The solid black circles represent data that are above the 90^th^ percentile or below the 10^th^ percentile for the finishing time for each city. There were no statistically significant differences in the winning times between the three cities (Kruskal-Wallis Analysis of Variance on Ranks, *p* = 0.872, *r* = 0.01 for all paired combinations).

**Fig 2:**
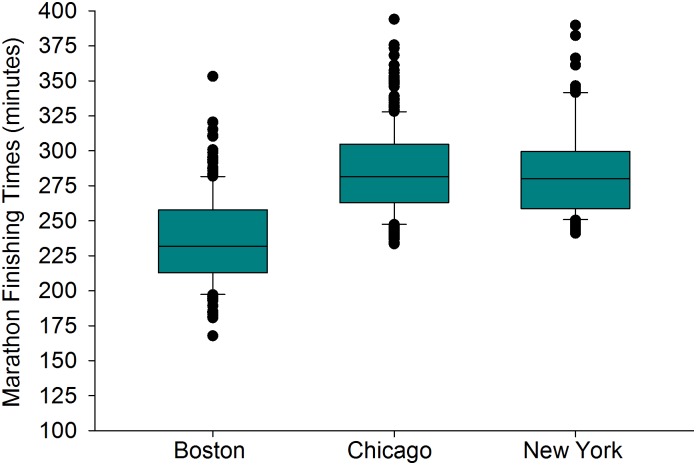
The median finishing times combined across all age categories and both sexes for Boston (n = 232), Chicago (n = 391), and New York (n = 96) City Marathons between 2001 and 2016. Each box represents the 25^th^ (bottom border) 50^th^ (middle border), and 75^th^ (top border) percentiles. The whiskers (error bars) above and below each box represent the 90^th^ and 10^th^ percentiles, respectively. The solid black circles represent data that are above the 90^th^ percentile or below the 10^th^ percentile, respectively. Overall, there were differences between the median times between each city (Kruskal-Wallis Analysis of Variance on Ranks, *p* < 0.001). Specifically, Boston has significantly faster median finishing time compared to Chicago and New York, respectively, by about 48–50 minutes (*p* < 0.001, *r* = 0.59 between Boston vs. Chicago, and *r* = 0.45 between Boston vs. New York). There is no difference in median finishing times between Chicago and New York City (adjusted significance, *p* = 0.11, *r* = 0.1).

**Fig 3:**
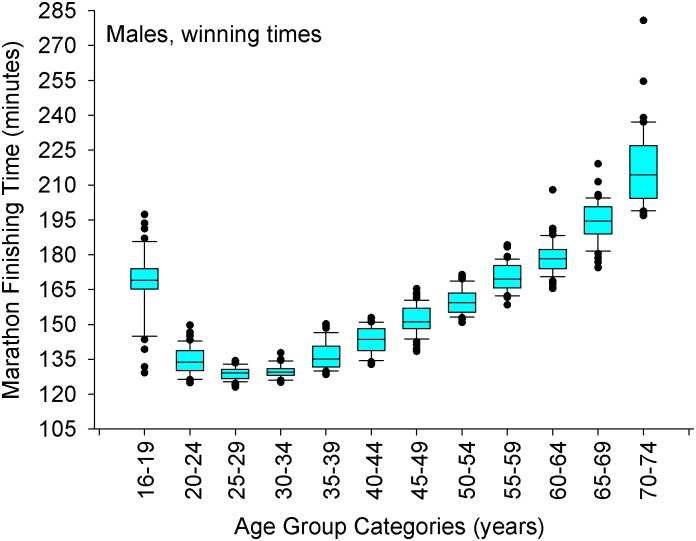
Marathon finishing times for the male winners in each age group category. The whiskers (error bars) above and below each box represent the 90^th^ and 10^th^ percentiles, respectively. Each box represents the 25^th^ (bottom border) 50^th^ (middle border), and 75^th^ (top border) percentiles. The solid black circles represent data that are above the 90^th^ percentile or below the 10^th^ percentile, respectively. Using a step-down procedure for multiple comparisons, mean times were similar between 25–34 years of age (*p* = 0.10 for a 2-sided test, adjusted significance, *p* = 0.48) and then increased at 35–39 years old age group. A Mann-Whitney test revealed significant differences between the 30–34 (n = 47) and 35–39 (n = 47) years old age groups (*p* < 0.01).

**Fig 4:**
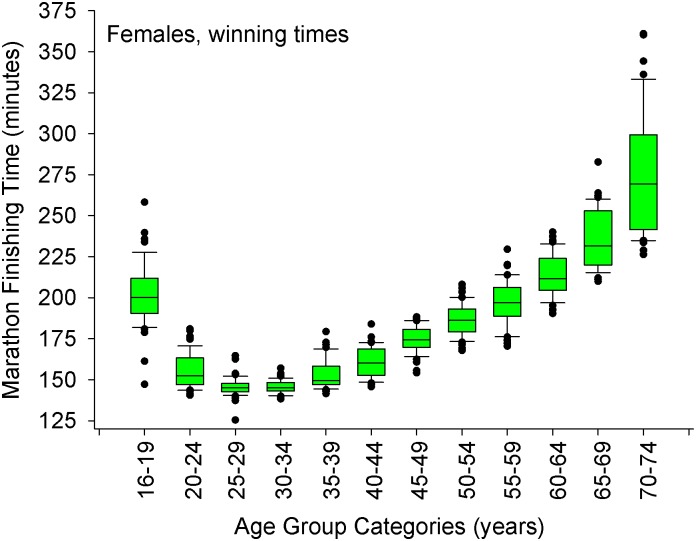
Marathon finishing times for the female winners in each age group category. The whiskers (error bars) above and below each box represent the 90^th^ and 10^th^ percentiles, respectively. Each box represents the 25^th^ (bottom border) 50^th^ (middle border), and 75^th^ (top border) percentiles. The solid black circles represent data that are above the 90^th^ percentile or below the 10^th^ percentile, respectively. When examining the four fastest age groups (20–24, 25–29, 30–34, 35–39 years of age), there were differences between the finishing times between the four age groups, overall (Kruskal-Wallis Analysis of Variance on Ranks, *p* < 0.001). Using a step-down procedure for multiple comparisons, mean times were similar between 25–34 years of age (*p* = 0.95 for the 2-sided test, adjusted significance, *p* = 1.00) and then increased at the 35–39 years old age group. A Mann-Whitney test revealed significant differences between the 30–34 (n = 47) and 35–39 (n = 47) years old age groups (*p* < 0.01).

**Fig 5:**
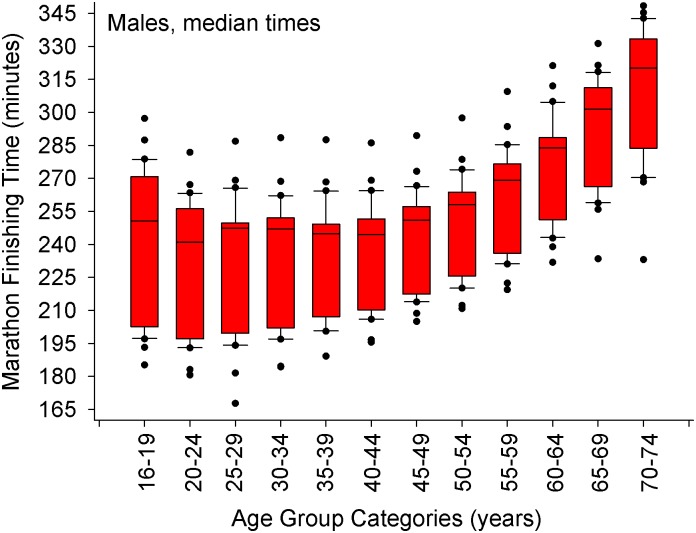
Median marathon finishing times for the males in each age group category. The whiskers (error bars) above and below each box represent the 90^th^ and 10^th^ percentiles, respectively. Each box represents the 25^th^ (bottom border) 50^th^ (middle border), and 75^th^ (top border) percentiles. The solid black circles represent data that are above the 90^th^ percentile or below the 10^th^ percentile, respectively. Overall, there was a difference in the median finishing times between groups (Kruskal-Wallis Analysis of Variance on Ranks, *p* < 0.001). Using a step-down procedure for multiple comparisons, median times were similar between 16–49 years of age (*p* = 0.39 for the 2-sided test, adjusted significance, *p* = 0.58) and then increased in the 50–54 age group and beyond.

**Fig 6:**
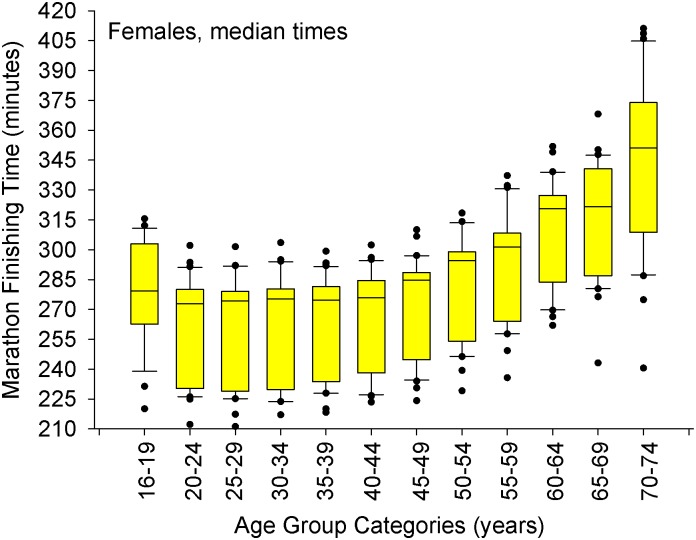
Median marathon finishing times for the females in each age group category. The whiskers (error bars) above and below each box represent the 90^th^ and 10^th^ percentiles, respectively. Each box represents the 25^th^ (bottom border) 50^th^ (middle border), and 75^th^ (top border) percentiles. The solid black circles represent data that are above the 90^th^ percentile or below the 10^th^ percentile, respectively. Using a step-down procedure for multiple comparisons, median times were similar between 24–49 years of age (*p* = 0.07 for the 2-sided test, adjusted significance, *p* = 0.14) and then increased in the 50–54 years old age group.

A small number of specific data points were not available in certain website databases, and thus could not be included in the analysis. The 2012 NYC marathon was canceled, so no data were available. Median finishing times for the NYC marathon were only available for four years (2013–2016), and median data for the Boston Marathon were available for ten years (2006–2016). This included winners of each age category between 2001 and 2016 [S1 Data (all ages, 1125 cases) and S2 Data (≥ 35 years old, 751 cases)] and the median finishers [S3 Data (all ages, 719 cases) and S4 Data (≥ 50 years old, 300 cases)**]**. A summary of the results for both age group winners (including overall champions) are presented in Figs 1, 3 and 5, and mean marathon performance per age group are presented in Figs 2, 4 and 6. Specifically, a comparison of performances between cities are depicted in Fig 1 (age group winners) and Fig 2 (median finishers in each age group).

### Overall champions of each marathon

The overall champions for each of the Boston, Chicago, and NYC marathons from 2001–2016 are presented in Tables 1 and 2. From 2001–2016, 47 marathons occurred from these cities. There were 9 men that were overall champions more than once [Cheruiyot (6), Mutai (3), Rop (2), Keflezighi (2), Desisa (2), Lel (2), Gomes Dos Santos (2), Rutto (2), Wanjiru (2)] and 11 women that were overall champions more than once [Jeptoo (5), Ndereba (4), Radcliff (4), Keitany (3), Okayo (3), Baysa (3), Kiplagat (2), Prokopcuka (2), Adere (2), Zakharova (2), Grigoryeva (2)]. Thus, 49% of the men’s marathons contained repeat winners, and 68% of the women’s marathons contained repeat winners. When comparing these two proportions, there was near significance (Chi-Square = 3.46, *p* = 0.063). The mean age (and SD) for the men’s and women’s winners for all marathons combined were 28.3 (3.7) years old (95% bootstrapped CI = 27.3 to 29.4 years old), and 30.8 (3.4) years old (95% bootstrapped CI = 29.9 to 31.8 years old), respectively. This age difference of 2.5 years (95% bootstrapped CI = 1.0 to 4.0 years) was statistically significant (*p* = 0.004), and was a medium effect (effect size = 2.5/3.8 = 0.66).

### Differences in finishing times between cities

There was no difference in finishing times for the winners of each age group between the three cities (Fig 1, *r* = about 0.01 for all paired combinations). However, there was a difference in the median finishing times for each age group between the three cities (Fig 2, *r* = 0.59 between Boston vs Chicago, and *r* = 0.45 between Boston vs. New York, and *r* = 0.1 between Chicago and New York). The median finishing times for the Boston Marathon were significantly faster compared to Chicago and New York, respectively, by about 48–50 minutes (*p* < 0.001). There was no statistical difference in median finishing times between Chicago and New York City (adjusted significance, *p* = 0.11). No data was eliminated from the analyses in Figs 1 or 2 (see S1 and S3 Data).

### Age-group winners

When the winners of each age group are assessed (n = ∼47 data points per age group, Table 3), the fastest times were found to be between 25–34 years old, and then the times became statistically slower beginning at 35 years of age (*p* < 0.001, Figs 3 and 4).

**Table 3:** Descriptive data for the female and male winning time for each age group using the combined data for Boston (2001–2016), NYC (2001–2016), and Chicago marathons (2001–2016).

Age group category (years)	Sample size	Mean (SD) age in years	Mean (SD) winning time in minutes	Median winning time in minutes	95% CI for the mean winning time in minutes	95% CI for the median winning time in minutes
Females						
16–19	46	18.9 (0.3)	201.5 (19.5)	200.2	196.4 to 207.2	194.0 to 205.2
20–24	47	22.9 (1.2)	155.3 (10.4)	152.3	152.4 to 158.2	149.4 to 159.1
25–29	47	27.3 (1.4)	145.7 (6.0)	145.2	143.9 to 147.4	144.3 to 147.0
30–34	47	32.0 (1.4)	145.7 (4.0)	145.1	144.4 to 146.8	144.4 to 146.1
35–39	47	36.6 (1.4)	153.0 (8.8)	149.5	150.5 to 155.8	148.4 to 152.8
40–44	47	41.1 (1.1)	160.5 (9.2)	160.2	157.9 to 163.3	154.3 to 165.2
45–49	47	46.2 (1.4)	174.8 (8.1)	174.4	172.5 to 177.6	171.6 to 178.0
50–54	47	51.5 (1.4)	186.6 (9.6)	186.3	183.7 to 189.2	183.7 to 189.2
55–59	47	56.4 (1.2)	197.1 (13.5)	197.1	193.1 to 201.3	192.9 to 201.1
60–64	47	60.9 (1.1)	213.3 (12.7)	212.4	209.8 to 217.0	209.4 to 217.1
65–69	47	65.9 (1.1)	235.1 (17.8)	231.6	230.0 to 240.3	229.1 to 239.9
70–74	46	71.0 (1.2)	276.8 (36.2)	269.4	266.7 to 287.1	264.4 to 287.2
Males						
16–19	46	18.7 (0.5)	167.7 (14.0)	169.0	163.7 to 171.7	164.0 to 172.3
20–24	47	22.9 (1.3)	134.2 (5.9)	133.7	132.6 to 138.9	132.2 to 135.7
25–29	47	27.2 (1.5)	128.9 (2.7)	129.0	128.2 to 129.8	128.1 to 129.8
30–34	47	31.7 (1.4)	130.0 (2.7)	129.5	129.2 to 130.7	129.1 to 130.7
35–39	47	36.9 (1.5)	136.4 (6.1)	135.1	134.7 to 138.2	134.3 to 138.0
40–44	47	41.1 (1.2)	143.3 (5.6)	144.0	141.6 to 145.0	141.6 to 145.2
45–49	47	46.6 (1.3)	152.2 (6.2)	151.2	150.6 to 154.0	150.5 to 154.0
50–54	47	51.0 (1.2)	159.9 (5.3)	159.5	158.4 to 161.4	158.3 to 161.3
55–59	47	56.2 (1.3)	169.8 (6.0)	169.3	168.1 to 171.5	167.8 to 171.4
60–64	47	61.0 (1.2)	178.3 (7.6)	177.9	176.1 to 180.4	176.0 to 180.0
65–69	47	66.2 (1.3)	194.4 (9.0)	194.5	191.9 to 196.9	191.9 to 196.9
70–74	47	71.2 (1.4)	217.1 (16.5)	214.5	212.7 to 221.8	208.0 to 219.8

The 95% CI is made from 1000 bootstrapped samples

As we were concerned with the rate of slow-down beginning at 35 years old, we initially examined 751 subjects between 35 and 74 years old that won their respective age group (this includes the overall champions) (S2 Data); however, 42 cases were eliminated due to the fact they were outliers (standard deviation of the residuals were ≥ 3.0. The outlying cases were 568–9, 571, 581–2, 585, 587, 591, 597, 599–601, 659, 661–5, 667–9, 674–81, 683, 685–6, 690–2, 697–9, 703, 721, 724, 727, Regression results (n = 709 data points ≥ 35 years old) indicated an overall model of two variables (age, sex) that significantly predicted winning marathon finishing time [adjusted r^2^ = 0.88, F(2,706) = 2539, SEE = 10 minutes 14 seconds, *p*<0.001, Eq 1]. The fit of the model was excellent (S1 Fig). The model accounted for 88% of the variance in winning marathon times at 35 years old up to 74 years old. About 70% of the variance in the model was accounted for by age, and ∼18% by sex. The 95% CI of the slope for the decrease in marathon time with age after 34 years old, was (min: sec) 2:14 to 2:23 per year. Overall, men were 23:37 to 26:39 faster compared to women (95% CI) beginning at 35 years old. A quadratic function did not enhance the fit beginning at 35 years old up to 74 years old.

Winning finishing time (min)=2.31⋅(age in years)−25.14⋅(sex)+69.49 (1)

(Sex: 0 = female, and 1 = male)

Furthermore, when comparing males versus females, the results demonstrate that female winners slow down at a faster rate than male winners beginning at 35 years old [women = 2:33 (min:sec) per year, 95% CI: 2:27 to 2:40, n = 336; men = 2:06, 95% CI: 2:02 to 2:11, n = 373] (*z* = 6.48, Df = 705, *p* < 0.001).

### Average finisher

As for the average man/woman finisher who finishes in the middle of his/her age group (n = 30 data points per age group, Table 4), the slowing of marathon times does not begin until 50 years old for both sexes (Figs 5 and 6).

**Table 4:** Descriptive data for the female and male median finishing time for each age group using the combined data for Boston (2001–2016), NYC (2001–2016), and Chicago marathons (2001–2016).

Age group category (years)	Sample size	Mean (SD) age in years	The mean (SD) of the “median” finishing times in minutes	The median of the “median” finishing times in minutes	95% CI for the mean of all “median” finishing times in minutes	95% CI for the median of all the “median” finishing times in minutes
Females						
16–19	29	18.5 (0.8)	278.8 (26.6)	279.4	269.2 to 288.1	270.9 to 298.0
20–24	30	23.0 (1.1)	262.7 (25.2)	272.9	252.8 to 271.9	256.7 to 277.9
25–29	30	27.3 (1.3)	262.6 (26.2)	274.3	252.7 to 272.6	257.0 to 278.8
30–34	30	31.9 (1.5)	263.5 (27.3)	275.4	254.1 to 273.5	261.2 to 279.5
35–39	30	36.8 (1.3)	264.2 (25.3)	274.7	254.8 to 273.3	255.1 to 280.8
40–44	30	42.0 (1.5)	266.5 (24.8)	275.9	257.0 to 275.8	251.8 to 281.7
45–49	30	46.4 (1.5)	273.4 (24.8)	284.6	264.4 to 282.6	275.0 to 287.1
50–54	30	51.7 (1.4)	283.4 (26.0)	294.5	273.2 to 292.6	277.3 to 297.3
55–59	30	56.8 (1.3)	293.7 (26.6)	301.5	284.0 to 303.5	292.6 to 305.2
60–64	30	61.3 (1.4)	310.3 (26.0)	320.6	300.4 to 319.4	299.1 to 324.0
65–69	30	66.3 (1.3)	315.9 (28.6)	321.6	306.0 to 326.6	304.8 to 330.9
70–74	30	71.6 (1.4)	340.8 (43.3)	351.1	325.0 to 355.8	316.9 to 361.2
Males						
16–19	30	18.4 (0.7)	242.7 (32.0)	250.7	230.7 to 253.9	234.7 to 260.6
20–24	30	22.7 (1.5)	232.8 (29.1)	241.1	222.7 to 243.3	219.7 to 251.1
25–29	30	27.6 (1.1)	233.3 (30.0)	247.4	222.2 to 244.5	231.3 to 249.6
30–34	30	31.8 (1.2)	234.7 (28.5)	247.1	224.7 to 244.9	232.0 to 249.5
35–39	30	37.0 (1.4)	235.7 (25.2)	244.9	225.9 to 244.7	228.3 to 248.3
40–44	30	41.6 (1.4)	237.6 (23.8)	244.5	229.5 to 245.9	237.7 to 250.5
45–49	30	46.6 (1.2)	243.5 (21.5)	251.1	236.1 to 251.5	244.4 to 254.2
50–54	30	51.8 (1.5)	251.0 (22.1)	258.1	243.4 to 258.4	253.2 to 262.2
55–59	30	56.5 (1.3)	262.7 (22.9)	269.2	254.5 to 270.5	263.7 to 274.5
60–64	30	61.6 (1.3)	277.2 (22.9)	283.9	268.8 to 285.1	281.4. to 288.1
65–69	30	66.6 (1.4)	293.6 (24.4)	301.6	284.3 to 301.6	294.0 to 307.8
70–74	30	71.1 (1.3)	310.9 (29.9)	320.1	299.8 to 321.2	300.6 to 329.0

The median time is the marathon time that is the 50^th^ percentile for the age group category. Mean (SD). The 95% CI is made from 1000 bootstrapped samples

As we were concerned with the rate of slow-down for the median finishers in each age group, we initially examined subjects between 50 and 74 years old that placed at the 50^th^ percentile for their group. There were 300 subjects between 50 and 74 years old that won their respective age group, but 10 data points were eliminated due to the fact they were outliers (standard deviation of the residuals were ≥ 3.0). These outliers were cases 186, 213, 242, 250, 252–3, 262–4, 266 (S4 Data). Regression results (n = 290 data points ≥ 50 years old) indicated an overall model of three variables (city, age, sex) that significantly predicted the median marathon finishing time [adjusted r^2^ = 0.86, F(3,286) = 608, SEE = 13 minutes 3 seconds, *p*<0.001, Eq 2]. The fit of the model was very good (S2 Fig). The model accounted for 86% of the variance in median finishing time at 50 years old up to 74 years old. About 40% of the variance in the model was accounted for by the city, ~29% by age, and ∼18% by sex. Since the median times were not different between Chicago or NYC (Fig 2), they were grouped together. The 95% CI of the slope for the decrease in marathon time with age beginning at 50 years old (min: sec) was 2:36 to 3:02. A quadratic function did not enhance the fit in the subjects beginning at 50 years old up to 74 years old.

Median finishing time (min)=46.96⋅(city)+2.81⋅(age) –29.77⋅(sex)+57.18 (2)

(For City: 1 = Boston, 2 = Chicago or NYC; for Sex 1 = male, 0 = female)

Furthermore, when comparing males versus females, the results demonstrate that the average female slows down similarly compared to the average male beginning at 50 years old [women = 2:36 (min:sec) per year, 95% CI: 2:16 to 2:56, n = 140; men = 2:57, 95% CI: 2:40 to 3:14, n = 150] (*z* = 1.60, Df = 286, *p* = 0.111).

## Discussion

The first aim of this study was to determine the age group at which marathon performance declines in top male and female runners and to compare that to the runners of average ability. Based on data from three of the largest marathons in the U.S., it was determined that fastest runners are at their best between 25 and 34 years of age, and statistically slow down at 35 years of age. In this large dataset, the mean age for the overall champions of the three marathons for men was 27.3 to 29.4 years old, and for the women was 29.9 to 31.8 years old (95% bootstrapped CI). These confidence intervals fall within previous reports of peak performance of the marathon and confirm that elite women tend to achieve peak performance 1–2 years later than elite men [23–25, 32, 33]. With regards to the male and female runners of average ability, performance declines were noticed beginning at 50 years old.

Another purpose of this of this study was to examine the age-related decline in marathon performance between age group winners and the average marathon finisher (the median running time in his/her age group). Age-group winning women runners slowed down at about 30 s per year faster rate compared to age group winning men runners in a linear fashion from 35 years of age to 74 years of age, while the average man and women finisher declines similarly (2 minutes and 47 s per year beginning at 50 years old up to 74 years old). This data is similar to Leyk *et al*. (2010) who demonstrated that when grouping all runners together (age group winners and all other runners), marathon times do not change until after 54 years old [15]. In fact, their first figure [15] is similar to Figs 5 and 6 of this paper. While the location did not matter for age-group winners, the Boston Marathon had faster median finishing times compared to NYC and Chicago because Boston has strict qualification standards. New York and Chicago marathons also have a guaranteed entry for those that meet a qualification time (based on age and sex), but most individuals that enter the Chicago or NYC marathons have entered into a random lottery draw, where no qualification time is needed. No random lottery draw exists for the Boston Marathon. Although city (or more appropriately, the qualifying standard) does influence the actual finishing time of the median finisher and therefore was appropriate to include in the model, its inclusion should not detract from the broader question regarding the influence of age and sex on declines in marathon performance.

This is the first study to concurrently examine more than a decade of age group winners and median finishers from multiple marathons. As such, the dataset obtained from these three marathons permitted the development of linear models superior to those within the existing literature. Indeed, this data is descriptive in nature. However, the analyses allow us to confirm a few interesting observations.

The first interesting observation was that about 70% of the decline in the winners’ finishing times beginning at 35 years old was due to aging while only about 20% was due to sex. Thus, compared to the physiological effects of aging, differences between sexes played a minor role in declining in marathon performance. This held true even for the average runners, where age was twice as important to the decrease in median finishing times compared to sex. However, the contribution of sex to declines in marathon performance is not necessarily solely due to physiological differences between men and women. Previous data has demonstrated that approximately 33% of the variation in marathon performance attributed to sex differences is partly due to a greater participation rate in men [12]. In other words, there may be plenty of women in the world who are physiologically capable of performances similar to the female winners in each age group, but a relative lack of actual participants in the marathon skews the average times of the top female competitors. This may be especially so for elite women, where the very best female runners may simply have less competition, which may alter strategy and influence finishing times. In 2015, 56% of U.S. marathon finishers were men, while 44% were women [34]. In this study, there was an 11% difference in marathon times between men and women at the age of peak performance (Table 3); thus about 3.7% of that 11% difference may be attributed to more men participating in the marathon compared to women [12]. In other words, our data demonstrates that 18% of the variation in marathon performance is due to a sex difference, but 6% of that 18% shared variance is due to greater male participants compared to female participants.

A second interesting observation was that the average man slows similarly compared to the average woman beginning at 50 years of age. This is in contrast to the age-group winners, where women slow at a ~30 s per year greater rate than men. We suspect that group differences in reproductive hormonal status may influence these differences. Indeed, menstrual dysfunction is not uncommon in young endurance athletes, with a reported prevalence of ~24% in distance runners [35], and heavy menstrual bleeding more prevalent in elite athletes [36]. It is possible that differences between reproductive hormone status between elite and recreational athletes may influence aging-related effects on performance. For instance, former post-menopausal endurance athletes have enhanced endothelial function compared to age-matched controlsd, but hormone replacement therapy counters this adaptation in this population [37]. Additionally, age-related changes in body composition may be different between sexes, and this may differ between competitive runners compared to recreational runners.

It is accepted that human physical performance declines with advancing age and there are multiple theories available to explain this mechanism besides the obvious reduction in aerobic capacity. Some of these theories include decreased motivation [26, 38], the decline in the magnitude of the training stimulus [39], and orthopedic issues [40]. In champion runners, the former two may occur but are unlikely to explain performance declines in the elite population, in particular for those professional runners whose financial income is dependent on fast finishing times.

### Limitations

The median finishing times for the NYC marathon were only available for four years (2013–2016), and median data for the Boston Marathon were available for ten years (2006–2016). Thus we do not have the complete 2001–2016 data set from these three marathons. However, we are confident that the use of these three marathons over several years provides us with enough data to give a careful analysis. The first figure in the article by Leyk and colleagues [15] is similar to Figs 5 and 6 of this study. Thus we are confident that our results are valid.

There may also be some concern that we eliminated a small percentage of the original data points during the development of the two equations. We considered these data as outliers which were not appropriate to include as part of a valid regression model. However, if all the data is analyzed and no outliers are eliminated from the model, we see that for the age group winners beginning at 35 years of age up to 74 years of age, women still slow down at a faster rate than men, but the rate is now ~60 s per year faster slow-down rate in women compared to men (instead of ~30 s per year difference between women and men). For the average finisher, the results do not change: men and women slow down at similar rates at ≥ 50 years old up to 74 years old. Because we included the city where the race was run in our regression model, there may be concern that our equation for the median finisher is specific to these three races. Although it is clear that qualifying standards for a marathon (i.e., Boston) can influence actual finishing times, the magnitude of decline is comparable across events. Additionally, our complete dataset is freely available, so that interested researchers can perform their modeling of the data.

## Conclusions

In conclusion, elite performance in the 35–39 years old age group is significantly slower compared to the fastest age groups (25–29 and 30–34 years old age groups), while average runners in the 50–54 years old age group experience a significant reduction in performance compared to younger age groups. At 35 years old, the rate of decline in male age group winners is about two minutes per year, whereas the rate of decline in women age group winners is about two minutes thirty seconds per year. Average runners can expect to decrease similarly with age, with males and females slowing down at a rate of about 2:49 per year beginning at 50 years old.

## Supporting information

S1 Data The SPSS data set for the age group winners (all ages, 1125 cases). (SAV)

S2 Data The SPSS data set for age group winners (≥ 35 years old, 751 cases). (SAV)

S3 Data The SPSS data set for the median finisher (all ages, 719 cases). (SAV)

S4 Data The SPSS data set for the median finisher (≥ 50 years old, 300 cases). (SAV)

S1 Fig Visual Fit of Eq 1 in PowerPoint. For the age-group winners: First, a histogram of the frequency of the data points versus the standardized residuals is plotted on slide 1. Slide 2 shows the probability-probability plot. Slide 3 shows the standardized residuals plotted against the standardized predicted values.
(PPTX)

S2 Fig Visual Fit of Eq 2 in PowerPoint. For the median finishers: First, a histogram of the frequency of the data points versus the standardized residuals is plotted on slide 1. Slide 2 shows the probability-probability plot. Slide 3 shows the standardized residuals plotted against the standardized predicted values.
(PPTX)
