# Supplementary figures and images for: Declines in marathon performance: Sex differences in elite and recreational athletes
Source: PLoS One. 2017 Feb 10;12(2):e0172121. doi: 10.1371/journal.pone.0172121 (PMC5302805; doi:10.1371/journal.pone.0172121)

## Slide 1
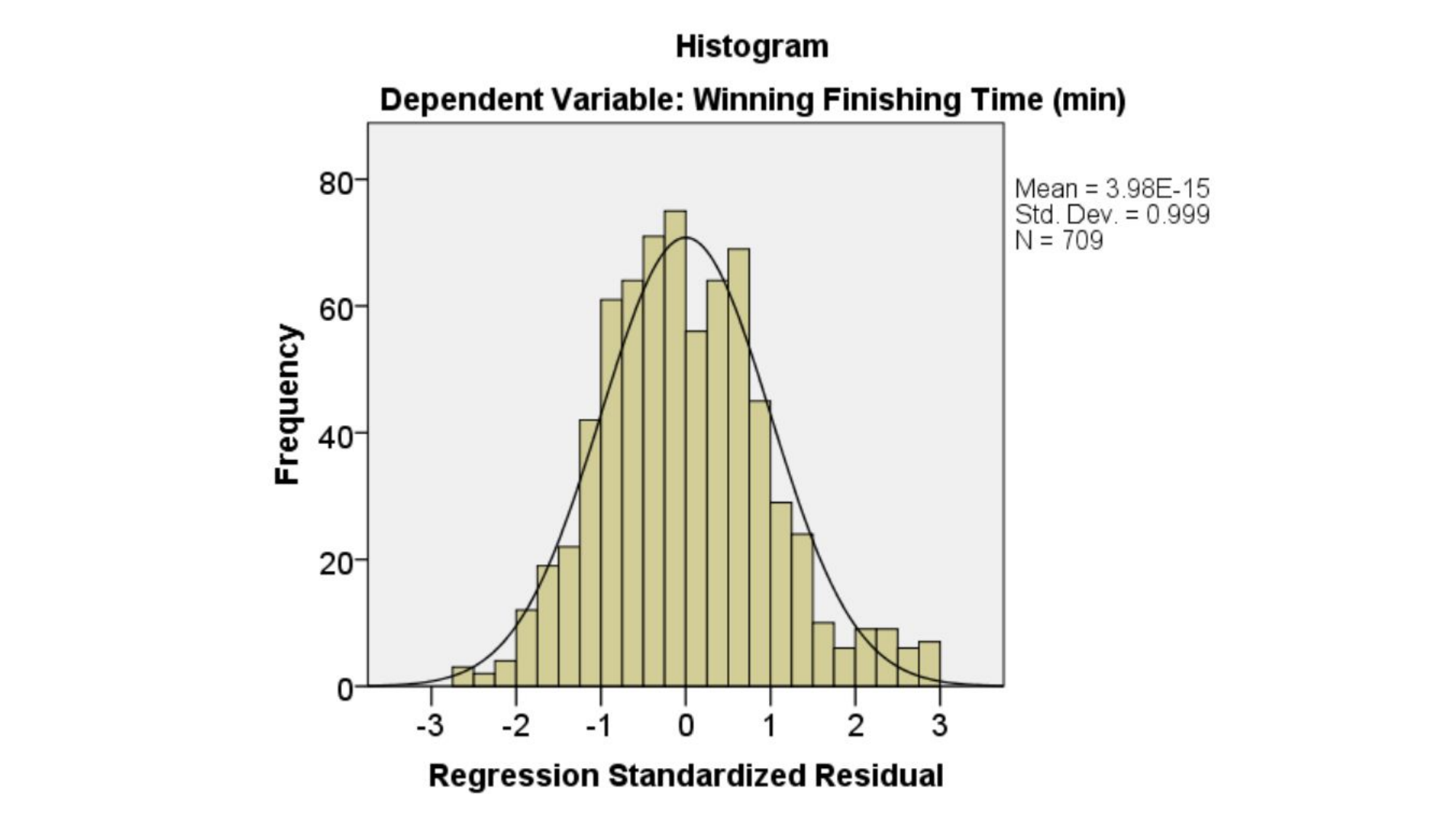

## Slide 2
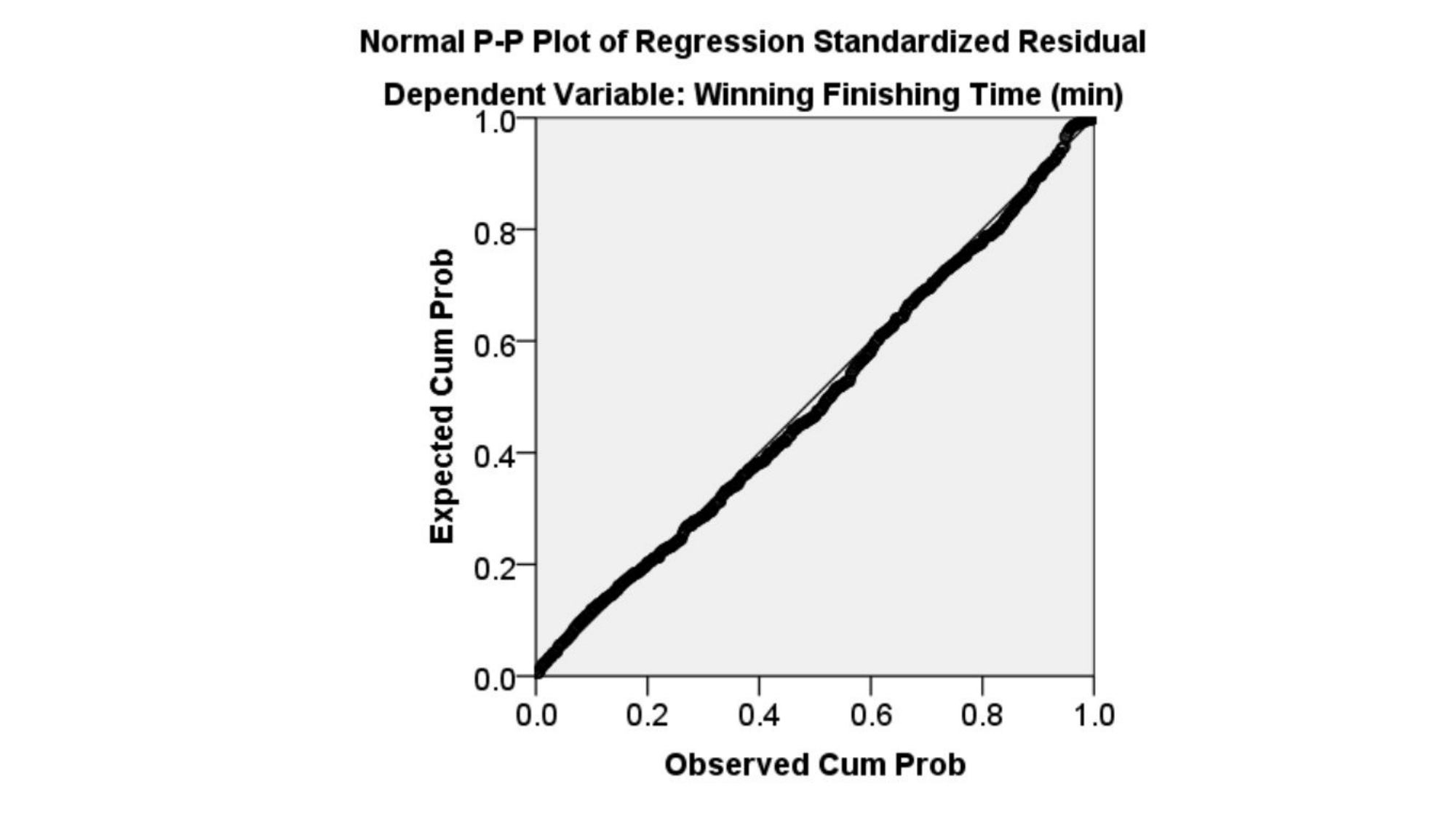

## Slide 3
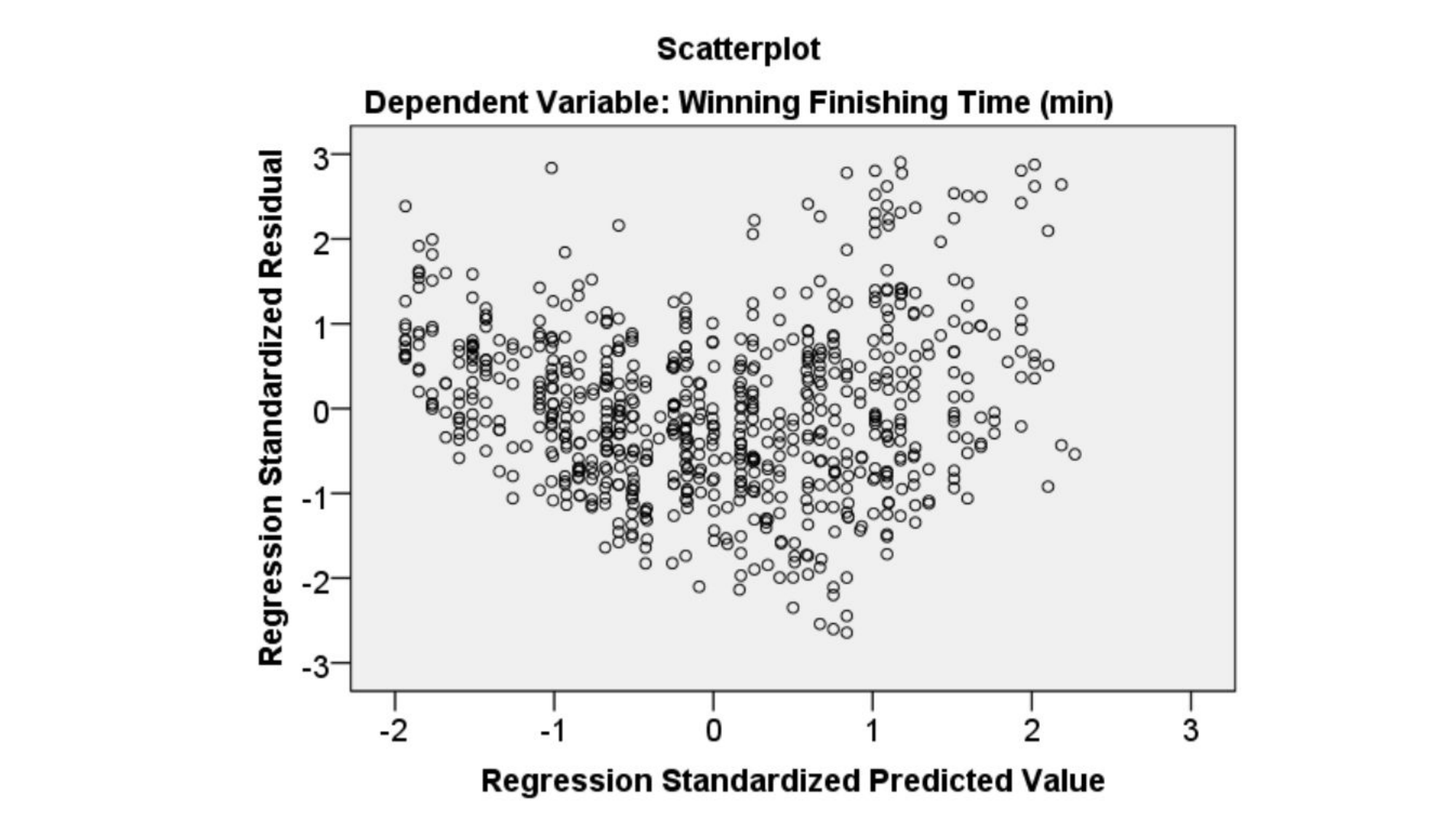

Supplement: S1 Fig — For the age-group winners: First, a histogram of the frequency of the data points versus the standardized residuals is plotted on slide 1. Slide 2 shows the probability-probability plot. Slide 3 shows the standardized residuals plotted against the standardized predicted values. (PPTX) [file pone.0172121.s005.pptx]

## Slide 1
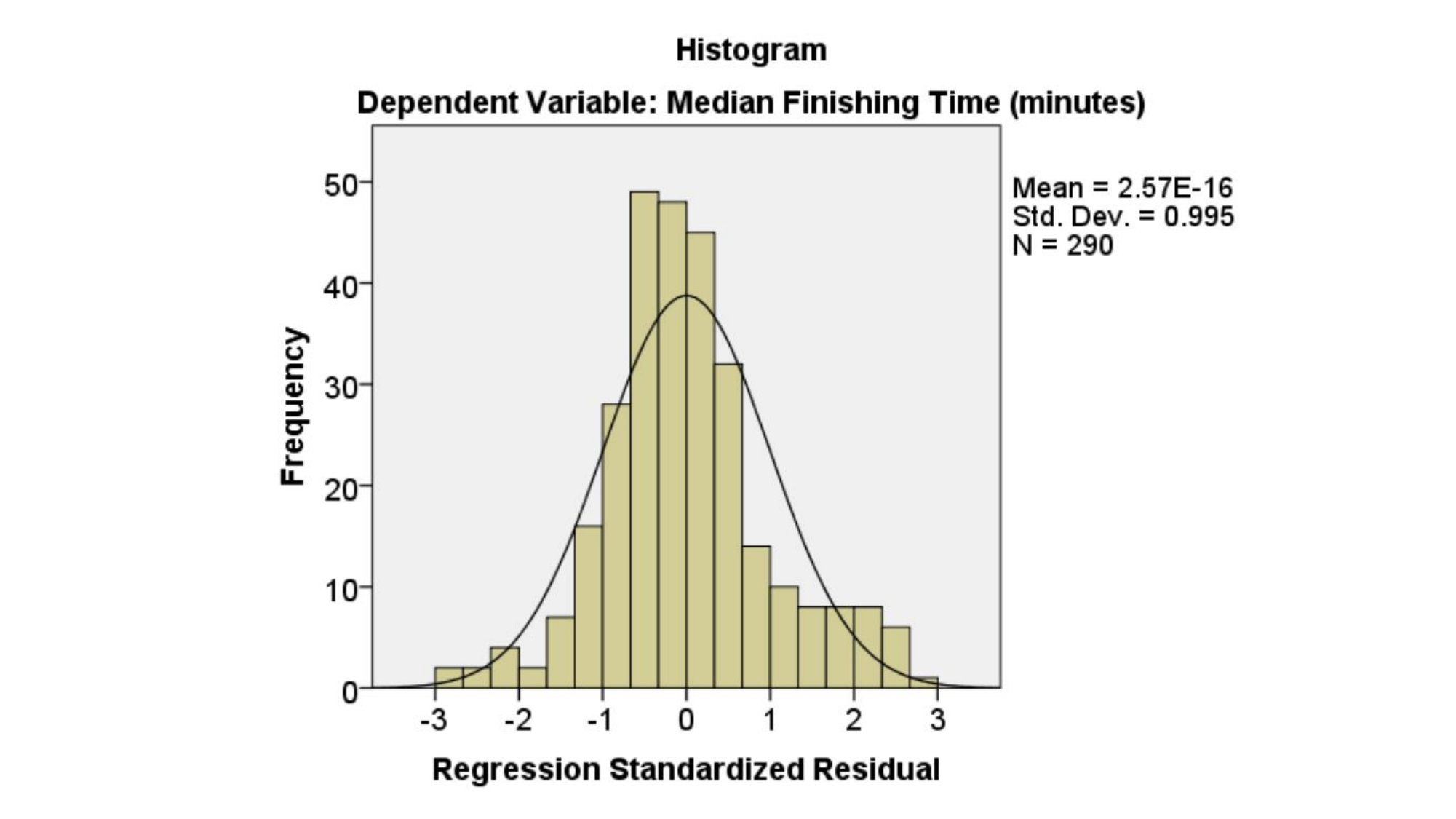

## Slide 2
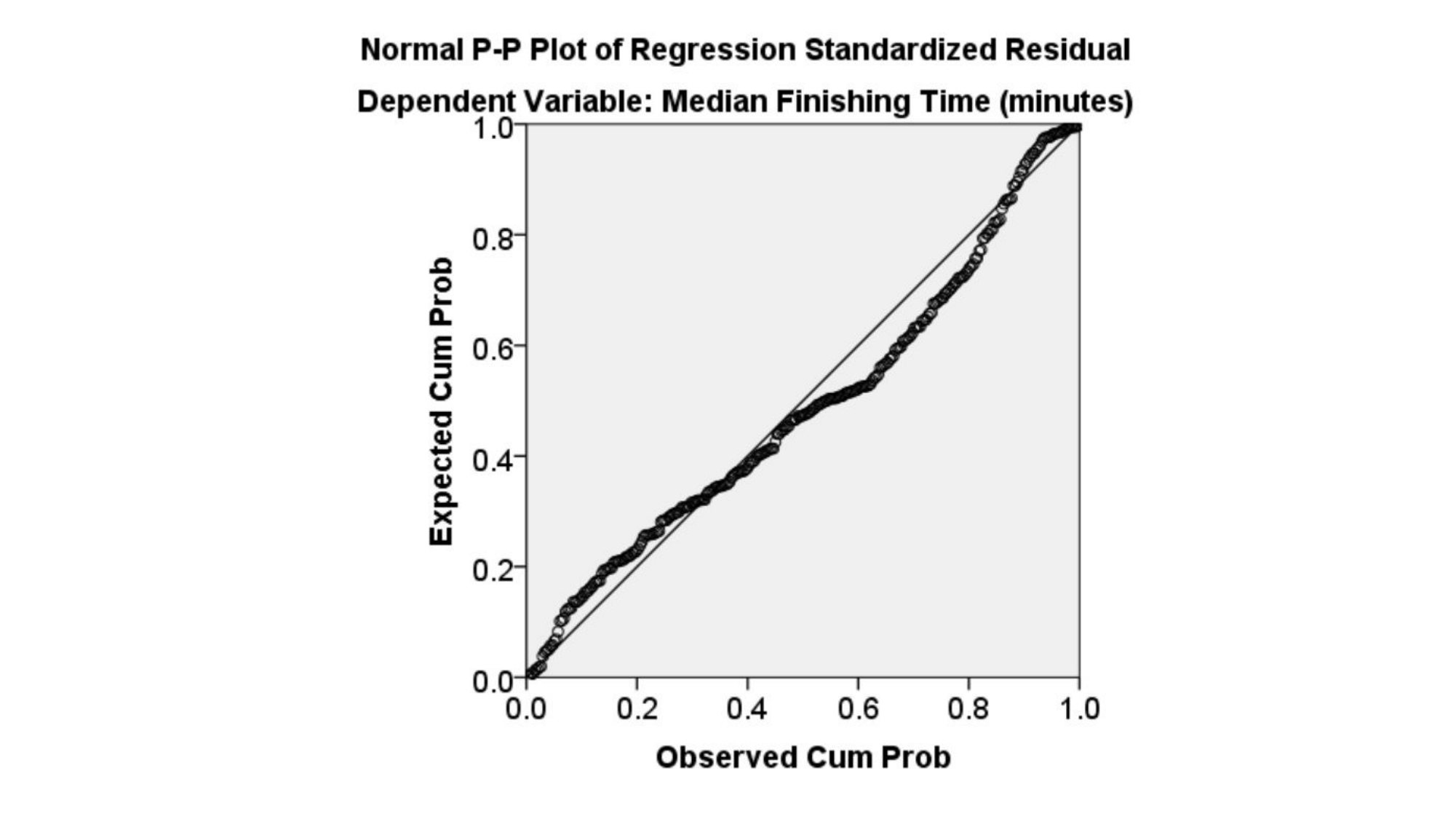

## Slide 3
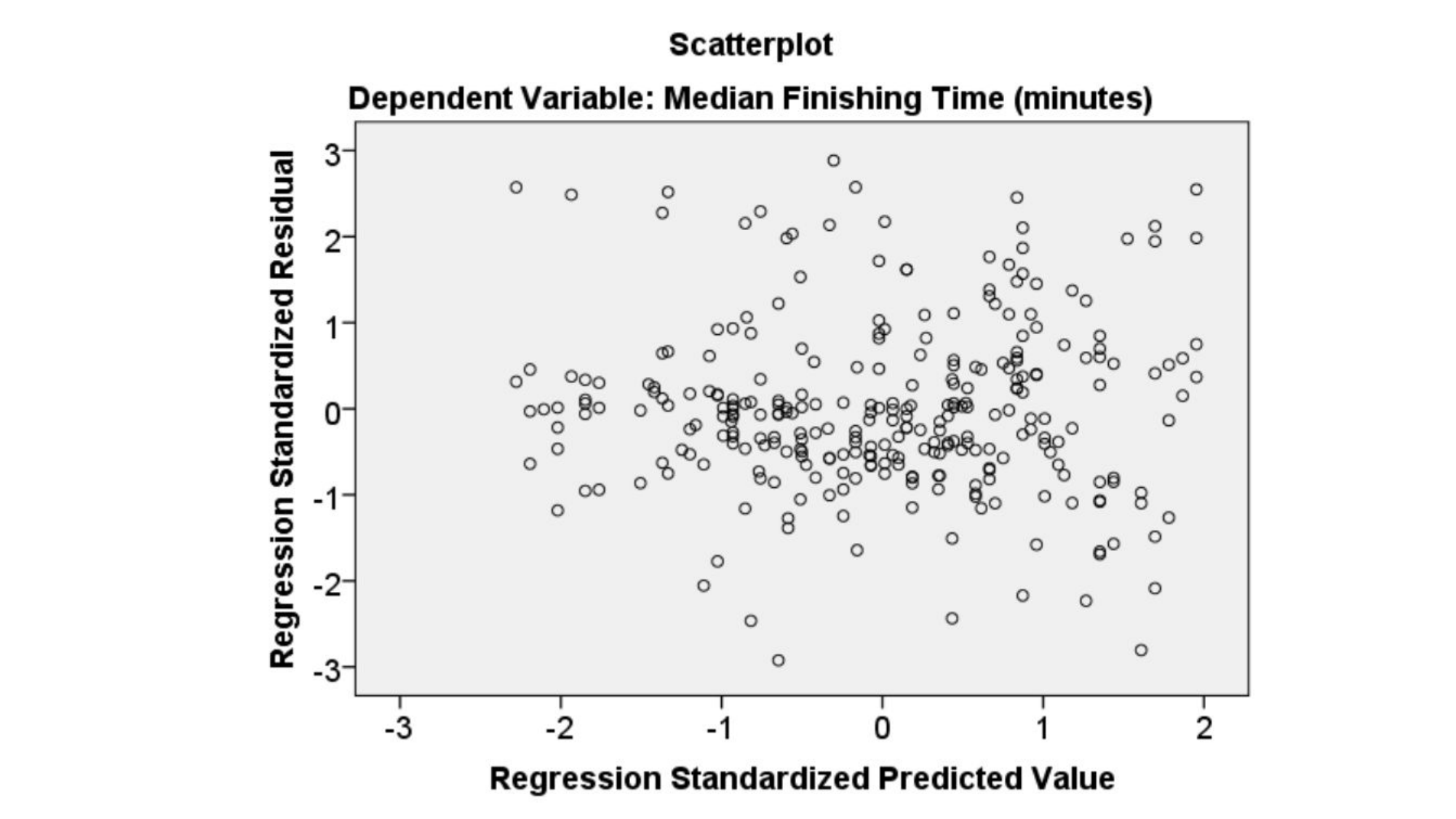

Supplement: S2 Fig — For the median finishers: First, a histogram of the frequency of the data points versus the standardized residuals is plotted on slide 1. Slide 2 shows the probability-probability plot. Slide 3 shows the standardized residuals plotted against the standardized predicted values. (PPTX) [file pone.0172121.s006.pptx]
